# HIV prevalence among 338,432 infertile individuals in Hunan, China, 2012-2018: A cross-sectional study

**DOI:** 10.1371/journal.pone.0238564

**Published:** 2020-09-10

**Authors:** Gang Liu, Huan Zhang, Wen-Bing Zhu, Yang-Qin Peng, Rui Ding, Meng-Lin Fan, Li-Qing Fan, Wei-Na Li

**Affiliations:** 1 The Institute of Reproduction and Stem Cell Engineering, School of Basic Medical Science, Central South University, Changsha, Hunan, China; 2 Reproductive and Genetic Hospital of CITIC Xiangya, Changsha, Hunan, China; 3 Clinical Research Center For Reproduction and Genetics In Hunan Province, Changsha, China; 4 Hunan Guangxiu Hi-tech Life Technology Co., Ltd., Changsha, China; University of Texas Health Science Center at San Antonio, UNITED STATES

## Abstract

**Background:**

The prevalence of human immunodeficiency virus (HIV) varies markedly among different risk groups in China, spreading fromhigh-risk populations to the general population. Indeed, China is in a critical period of HIV/acquired immunodeficiency syndrome (AIDS) prevention and control; however, data regarding HIV testing, infection and coinfection among infertile couples are lacking. This study aimed to estimate the HIV/AIDS prevalence to identify risk factors among infertile couples in Hunan, China.

**Methods:**

A cross-sectional hospital-based study was conducted to evaluate the prevalence of HIV/other infections (hepatitis B virus (HBV), hepatitis C virus (HCV), syphilis, and *Chlamydia trachomatis*, *Neisseria gonorrhoeae*, and *Mycoplasma genitalium* (MG) infections) among 338,432 infertile individuals in Hunan, China, from 2012 to 2018. We calculated linear trends in prevalence using bivariate linear regression.

**Results:**

The overall prevalence rates of HIV, chlamydia, gonorrhea, MG, syphilis, and HBV and HCV antibody positivity in this study were 0.04%, 1.73%, 0.05%, 2.60%, 2.15%, 12.01% and 0.56%, respectively. The predominant infection was HBV, followed by MG, syphilis, and chlamydia. Only 1.13% of the participants (382/338432) reported sexually transmitted disease (STD) signs and symptoms suggesting genital tract infection. However, from 2012–2018, the variation in HIV prevalence was not significant (β = 0.000, *P*_TREND_ = 0.907). The characteristics of the HIV-infected infertile population have not shifted dramatically, with women accounting for 32.56% of HIV cases in China. Overall, 87.60% of HIV-infected individuals have a relatively low education. In total, 37.98% of HIV-positive patients engage in high-risk behaviors.

**Conclusions:**

This study expands upon existing knowledge of HIV prevalence in the infertile Chinese population. However, much work is needed to achieve popularization of prevention knowledge and change concept. Routine HIV screening is urgently needed for all adults with high-risk behaviors.

## Introduction

The United Nations acquired immunodeficiency syndrome (AIDS) estimates that by the end of 2018, 37.9 million people with human immunodeficiency virus (HIV)/AIDS had survived globally, 1.8 million new HIV infections occurred that year, and 21.7 million were receiving highly active antiretroviral therapy (HAART)[1, 2]. Since 1985, when China reported its first case of AIDS, the disease has gradually spread, increasing at a rate of 30% per year. The National Health Care Commission suggested that by the end of 2018, China cumulatively reported that 1,250,000 people were living with diagnosed HIV infection[3]of which female patients accounted for 24.39%. It is estimated that 80,000 new infections occur every year in China[4]. At present, AIDS has become an important public health problem that seriously threatens the public health in China. Early sexually transmitted disease (STD) testing, treatment and prevention services are an important part of HIV medical strategies and can have significant public health benefits[5, 6].

Globally, much research has been directed at identifying the burden of HIV infection among higher risk populations, and HIV epidemics show marked variation among different risk groups in China. Many studies have also revealed the prevalence of HIV among the general population(0.1%), people who inject drugs (PWID)(10.5%), Chinese men who have sex with men (MSM, 7.3%), female sex workers (FSWs, 0.2%), and transgender women (14.8%)[7–16].

However, in the past 35 years, there have been significant changes in the HIV infection epidemic in China, which was initially exclusively among PWID; outbreaks due to plasma collection pollution occurred in the mid-1990s, and currently, transmission is almost completely via sexual contact[17, 18]. The 2018 Chinese National Guidelines for HIV/AIDS Diagnosis and Treatment has been the third update of the guidelines within 10 years[19]. Although the HIV epidemic among PWID has been well managed, the growing HIV epidemic through sexual contact has become more complex and even more difficult to control. The HIV/AIDS epidemic in China has spread from high-risk populations to the general population[3]. To tailor prevention programs for distinct populations, local epidemiological data are necessary.

An infertile couple is defined as a couple who had not had a positive pregnancy experience after one-year of regular unprotected sexual intercourse without contraceptive drugs use or other methods. They represent a distinct group characterized by sexually active individuals with a risky sexual behavior profile (e.g., never use condoms). Several social science studies have shown that infertility leads to the practice of “looking elsewhere for offspring”, especially among men and to a lesser extent women, and to unstable sexual relationships[20–22]. These behaviors in turn predispose women and men to HIV infection and sexually transmitted infections (STI)s. Risky sexual behavior puts women, and to a lesser extent men, at risk for infertility and is perpetuated or might be reinforced after they have become infertile, closing a vicious circle of risky sexual behavior, HIV infection and infertility. Couples in infertile relationships may constitute an important vulnerable group for HIV infection and may fuel the HIV epidemic.

A number of studies have shown an association between infertility and HIV infection, focusing mainly on reduced fecundity among HIV-positive people compared with HIV-negative people. HIV-positive people have lower conception rates and higher rates of pregnancy loss, which are thought to be due to greater susceptibility to pelvic inflammatory disease with resultant tubal factor infertility and to weight loss related anovulation, amenorrhea, male hypogonadismand impaired spermatogenesis[23–26]. HIV and possibly antiretroviral therapy may be associated with borderline semen abnormalities including low sperm count, low motility, and low volume[25, 27, 28].

However, few studies have examined the HIV prevalence and coinfection in infertile patients. This study aimed to estimate the HIV/AIDS prevalence and trends to identify factors associated with HIV among infertile couples in Hunan, China.

## Materials and methods

### Study area and population

Hunan is a landlocked province that is located in south eastern China. This study was conducted in Changsha, the capital of Hunan Province, which is known for its media and entertainment industries. By the end of 2018, there were 68,988,000 inhabitants in Hunan, and the urbanization rate was 56.02%. Among the permanent population, 51.54% were male, and 48.46% were female. Han individuals account for 89.9% of the total population, and the minority population accounts for 10.1%. There is a large population comprising ethnic minorities, including Tujia, Miao, Dong, Yao, Bai, Hui, Zhuang, Mongolian, Manchu and Uygur.

In 1989, the Chinese government formed a complete AIDS prevention and control monitoring network. Regulations and measures on AIDS prevention and control in Hunan Province have been in effect since May 1, 2012. China promotes "voluntary counseling and testing of AIDS”, and "medical staff provide AIDS counseling and testing on their own initiative”. For HIV/AIDS patients, medical staff should report the epidemic situation to the local center for disease control (CDC) in a timely manner and take corresponding measures in accordance with the Law of the People's Republic of China on the Prevention and Control of Infectious Diseases. The Chinese government implemented “four exemptions and one care “for AIDS patients, providing free anti-HIV drugs, free anonymous testing, free mother-to-child interruption, free schooling for AIDS orphans, and care for the elderly, orphans and widows.

The Reproductive and Genetic Hospital of CITICXiangya, in Changsha, Hunan, is a well-known reproductive hospital in China, which completed more than 50,000 in vitro fertilization (IVF) treatment cycles in 2018. Infertile couples are routinely screened for HIV before assisted reproductive therapy.

### Study design

This cross-sectional hospital-based study evaluated the prevalence of HIV/other sexually transmitted diseases (STDs) among 338,432 infertile couples who visited the outpatient department of the Reproductive and Genetic Hospital of CITICXiangya for prepregnancy examination between January 2012 and December 2018. We extracted relevant demographic and clinical information from all medical record data on January 2019. The patients were enrolled for treatment atour hospital following failure to achieve pregnancy after at least 1 year of unprotected sexual intercourse. The width of the defined age groups was designed to be equal among the five age groups (<20, 20–29,30–39,40–49 and ≥50 years of age). The present study, conducted in a single center in China, was approved by the Ethics Committee of the Reproductive and Genetic Hospital of CITIC-Xiangya (No. LL-SC-2018-056). We had no access to information that could identify individual participants during or after data collection.

### Inclusion and exclusion criteria for the infertile population in this study

Infertile individuals who visited the outpatient department of our hospital between 2012 and 2018 were included. The following exclusion criteria were applied: duplicate records; incomplete information records; significant abnormality records; refusal of counseling and testing for HIV/other infections; and lack of completion of all study procedures.

### Study procedures

All participants were counseled by physicians about sociodemographic and medical questions, regardless of the decision to undergo IVF. STD-related symptoms were measured by asking whether the participants experienced any of the listed symptoms (burning pain when urinating, genital discharge, ulcer/sores on penis/anus/genitals) in the last year. After the survey, the physicians performed physical examinations on each participant. For males, meatus urinarius secretions and semen and blood specimens were collected; for females, cervical secretions and blood samples were collected. HIV/other STD testing included HIV, hepatitis B virus (HBV), hepatitis C virus (HCV), syphilis(TP), *Chlamydia trachomatis* (CT), *Neisseria gonorrhoeae* (NG) and *Mycoplasma genitalium* (MG) testing. HIV-positive participants were administered a structured questionnaire in private by our trained hospital infection staff to collect information on sexual behaviors and condom use with various partners, any STD-related symptoms in the past six months, HIV/STD risk perceptions and drug use history using face-to-face or telephone interviews. Illicit drug use was defined as recreational use of a prohibited or controlled drug.

### Laboratory methods

Blood specimens were tested for antibodies against HIV, HBV, HCV, and TP. The HIV antibody test (Livzon, China) and the HIV 1/2 antibody test were used to confirm an HIV-positive result (Abon Biopharm, Hangzhou, China). The sample was considered HIV positive only if both tests were positive. Those with confirmed positive tests were referred to Hunan Provincial Center for Disease Prevention and Control for further confirmation and reports. HBV testing was performed with a diagnostickit (Abbott, USA) using standard assays. We defined past or present HBV infection as the presence of the total hepatitis B core antibody (HBcAb) and chronic HBV infection (CHBI) as the presence of both the total HBcAb and hepatitis B surface antigen (HBsAg). In this study, subjects with HbsAg-positive results were considered to have HBV infection. HCV antibody testing was performed with an ELISA diagnostic kit to detect antibodies against HCV(Livzon, China). Rapid plasma regain (RPR) (circle card tests for the syphilis TRUSTkit, Rongsheng Biotechnical Company, Shanghai, China) was used as a screening test, and positive samples were confirmed by a *Treponema palladium* particle agglutination assay (TPPA)(passive particle agglutination test for the detection of TP, Serodia, Fujirebio, Fuji, Japan). Subjects with positive results for both RPR and TPPA were considered to have a current TP infection. Male meatus urinarius secreta and female cervical secreta were collected for CT and NG testing by colloidal gold assays (Chemture, MaxMed Laboratories Inc., Shanghai, China), culture and Gram staining with chocolate agar plate culture (Huankai, Guangdong, China). Semen and vaginal swab specimens were tested via real-time probe simultaneous amplification and testing (SAT) (Rendu, Shanghai, China) for MG. The target gene 16S ribosomal RNA (rRNA) of MG was isolated and reverse transcribed to generate cDNA fragments. The results were interpreted as positive when the cycle threshold (Ct) was ≤35 and an absorption peak was observed in the melting curve.

### Statistical analysis

Analyses were descriptive and performed using IBM SPSS Statistics (v. 19.0; IBM Corp., Armonk, NY, USA). Baseline characteristics are presented as frequencies (%) for categorical data. The prevalence of HIV/STD infection with 95% confidence intervals (CIs) was calculated. We calculated linear trends in prevalence for 2012–2018 using bivariate linear regression. Beta coefficients for the year represent the average percentage point change (divided by 100) from one year to the next. Risk factors were assessed using chi-square or binary logistic statistics for categorical variables. All of the tests were two-tailed, and p values <0.05 were interpreted as statistically significant.

## Results

### Demographic characteristics

A total of 338,432 infertile individuals were included between January 1, 2012, and December 31, 2018. The characteristics of the study population with regard to sex, age, education and income are shown in Table 1.

**Table 1:** Characteristics of infertile population in China from 2012-2018.

Characteristic	n(%)							
	2012	2013	2014	2015	2016	2017	2018	2012–2018
Sex								
Male	15705(43.91)	17776(44.23)	19730(44.48)	21685(44.59)	24714(44.58)	25548(45.15)	26463(46.05)	151621(44.80)
Female	20060(56.09)	22418(55.77)	24629(55.52)	26945(55.41)	30727(55.42)	31034(54.85)	30998(53.95)	186811(55.20)
Age, y								
<20	3(0.01)	5(0.01)	4(0.01)	13(0.03)	22(0.04)	18(0.03)	21(0.04)	86(0.03)
20–29	11905(33.29)	12913(32.13)	14768(33.29)	17529(36.05)	17965(32.40)	17454(30.85)	17155(29.86)	109689(32.41)
30–39	20021(55.98)	22304(55.49)	23650(53.31)	24799(51.00)	28055(50.60)	29396(51.95)	30867(53.72)	179092(52.92)
40–49	3734(10.44)	4784(11.90)	5657(12.75)	6016(12.37)	8957(16.16)	9272(16.39)	8901(15.49)	47321(13.98)
≥50	102(0.29)	188(0.47)	280(0.63)	273(0.56)	442(0.80)	442(0.78)	517(0.90)	2244(0.66)
Education								
Never attended school	0	0	0	0	0	0	0	0
Primary school	3397(9.50)	3497(8.70)	3948(8.90)	5106(10.50)	3537(6.38)	4815(8.51)	3568(6.21)	27868(8.23)
Junior middle school	7221(20.19)	7062(17.57)	9821(22.14)	9556(19.65)	11615(20.95)	10434(18.44)	9596(16.70)	65305(19.30)
Senior school/technical Secondary school	17350(48.51)	20189(50.23)	21807(49.16)	24923(51.25)	28757(51.87)	28489(50.35)	29702(51.69)	171217(50.69)
College or above	7797(21.80)	9446(23.50)	8783(19.80)	9045(18.60)	11532(20.80)	12844(22.70)	14595(25.40)	74042(21.88)
Income(RMB/month)								
<5000*	21173(59.20)	23594(58.70)	25418(57.30)	27622(56.80)	30770(55.50)	31686(56.00)	30857(53.70)	191119(56.47)
≥5000	14592(40.80)	16600(41.30)	18941(42.70)	21008(43.20)	24671(44.50)	24896(44.00)	26604(46.30)	147313(43.53)
STD-related symptoms								
Yes	512(1.43)	635(1.58)	690(1.56)	548(1.13)	456(0.82)	437(0.77)	546(0.95)	3824(1.13)
No	35253(98.57)	39559(98.42)	43669(98.44)	48082(98.87)	54985(99.18)	56145(99.23)	56915(99.05)	334608(98.87)
Total	35765	40194	44359	48630	55441	56582	57461	338432

### HIV/other infection prevalence

The overall prevalence rates of HIV, CT, NG,MG,TP, HBV and HCV antibody positivity in this study were 0.04%,1.73%,0.05%,2.60%,2.15%,12.01% and 0.56%, respectively(Table 2). The predominant infection was HBV (21933/151621 (14.47%) males, 18704/186811 (10.01%)females), followed by MG(1407/56237 (2.50%) males, 1803/67373 (2.68%)females), TP(2268/151621 (1.50%) males, 5009/186811 (2.68%)females), and CT(1447/151621 (0.95%) males, 4422/186811 (2.37%) females). Of those participating, 16.65% (56336/338432) had at least one positive test, and 0.59% (1999/338432) had more than one positive test. The male HIV prevalence (0.06%, 87/338432) was higher than the female HIV prevalence (0.02%,42/338432). Only 1.13% of the participants (3824/338432) reported STD signs and symptoms suggesting genital tract infection. Most infertile participants in this study did not perceive any personal risk for HIV (99.99%,338,426/338,432). Only 4 participants admitted to having been tested for HIV in the past year, and2 infertile men reported prior history of HIV, using in vitro fertilization with donor semen(AID) in our hospital.

**Table 2:** Prevalence of HIV/other infections^a^ among infertile population in China from 2012-2018.

Infections/Sex	n(%)								β_TREND_	*P* value
	2012	2013	2014	2015	2016	2017	2018	2012–2018		
Male										
n	15705	17776	19730	21685	24714	25548	26463	151621		
HIV	8(0.05)	17(0.10)	6(0.03)	11(0.05)	13(0.05)	8(0.03)	24(0.09)	87(0.06)	0.000	0.936
CT	257(1.64)	191(1.07)	132(0.67)	229(1.06)	256(1.04)	216(0.85)	166(0.63)	1447(0.95)	-0.001	0.073
NG	5(0.03)	6(0.03)	5(0.03)	4(0.02)	6(0.02)	8(0.03)	2(0.01)	36(0.02)	0.000	0.107
MG	ND	ND	ND	ND	106(2.51)	645(2.52)	656(2.48)	1407(2.50)	0.000	0.563
TP	338(2.15)	333(1.87)	358(1.81)	414(1.91)	450(1.82)	188(0.74)	187(0.71)	2268(1.50)	-0.002	0.013
HBV	2501(15.92)	2901(16.32)	2971(15.06)	3131(14.44)	3512(14.21)	3475(13.60)	3442(13.01)	21933(14.47)	-0.005	<0.001
HCV	120(0.76)	137(0.77)	130(0.66)	144(0.66)	157(0.64)	147(0.58)	175(0.66)	1010(0.67)	0.000	0.031
Any infection^b^	3092(19.69)	3385(19.04)	3528(17.88)	3657(16.86)	4152(16.80)	4387(17.17)	4419(16.70)	26620(17.56)	-0.005	0.007
>1 infection	132(0.84)	97(0.55)	53(0.27)	73(0.34)	97(0.39)	210(0.82)	129(0.49)	791(0.52)	0.000	0.781
Female										
n	20060	22418	24629	26945	30727	31034	30998	186811		
HIV	3(0.01)	6(0.03)	7(0.03)	8(0.03)	5(0.02)	5(0.02)	8(0.03)	42(0.02)	0.000	0.982
CT	1013(5.05)	811(3.62)	947(3.85)	415(1.54)	492(1.60)	405(1.31)	339(1.09)	4422(2.37)	-0.007	0.003
NG	17(0.08)	13(0.06)	18(0.07)	22(0.08)	22(0.07)	24(0.08)	10(0.03)	126(0.07)	0.000	0.240
MG	ND	ND	ND	ND	138(2.58)	656(2.11)	1009(3.26)	1803(2.68)	0.003	0.602
TP	712(3.55)	739(3.30)	810(3.29)	837(3.11)	977(3.18)	425(1.37)	509(1.64)	5009(2.68)	-0.003	0.016
HBV	2302(11.48)	2426(10.82)	2593(10.53)	2640(9.80)	2937(9.56)	2872(9.25)	2934(9.47)	18704(10.01)	-0.004	0.001
HCV	133(0.66)	114(0.51)	103(0.42)	117(0.43)	148(0.48)	124(0.40)	146(0.47)	885(0.47)	0.000	0.123
Any infection	4833(24.09)	3925(17.51)	4289(17.41)	3890(14.44)	4137(13.46)	3908(12.59)	4734(15.27)	29716(15.91)	-0.014	0.030
>1 infection	334(1.67)	182(0.81)	91(0.37)	145(0.54)	139(0.45)	103(0.33)	214(0.69)	1208(0.65)	-0.001	0.125
Infected spouse										
HIV	0	3(0.48)	0	1(0.18)	0	0	2(0.29)	6(0.14)	0.000	0.935
CT	15(2.28)	4(0.63)	6(1.07)	10(1.82)	7(1.19)	5(0.71)	6(0.86)	53(1.21)	-0.001	0.249
NG	1(0.15)	2(0.32)	0	0	1(0.17)	1(0.14)	0	5(0.11)	0.000	0.360
MG	ND	ND	ND	ND	23(15.70)	131(18.58)	154(22.03)	308(20.30)	0.034	ND
TP	129(19.63)	124(19.68)	92(16.40)	85(15.48)	108(18.43)	14(1.99)	39(5.58)	591(13.47)	-0.027	0.026
HBV	319(48.55)	387(61.43)	324(57.75)	340(61.93)	378(64.51)	345(48.94)	352(50.36)	2445(55.73)	-0.005	0.755
HCV	7(1.07)	12(1.90)	6(1.07)	10(1.82)	8(1.37)	11(1.56)	7(1.00)	61(1.39)	0.000	0.797
Any infection	657(100.00)	630(100.00)	561(100.00)	549(100.00)	586(100.00)	705(100.00)	699(100.00)	4387(100.00)	ND	ND
>1 infection	9(1.37)	5(0.79)	2(0.36)	4(0.73)	3(0.51)	10(1.42)	6(0.86)	39(0.89)	0.000	0.958
Total										
HIV	11(0.03)	23(0.06)	13(0.03)	19(0.04)	18(0.03)	13(0.02)	32(0.06)	129(0.04)	0.000	0.907
CT	1270(3.55)	1002(2.49)	1079(2.43)	644(1.32)	748(1.35)	621(1.10)	505(0.88)	5869(1.73)	-0.004	0.001
NG	22(0.06)	19(0.05)	23(0.05)	26(0.05)	28(0.05)	32(0.06)	12(0.02)	162(0.05)	0.000	0.142
MG	ND	ND	ND	ND	244(2.55)	1301(2.30)	1665(2.90)	3210(2.60)	0.006	0.009
TP	1050(2.94)	1072(2.67)	1168(2.63)	1251(2.57)	1427(2.57)	613(1.08)	696(1.21)	7277(2.15)	-0.003	0.014
HBV	4803(13.43)	5327(13.25)	5564(12.54)	5771(11.87)	(11.63)	6347(11.22)	6376(11.10)	40637(12.01)	-0.004	<0.001
HCV	253(0.71)	251(0.62)	233(0.53)	261(0.54)	305(0.55)	271(0.48)	321(0.56)	1895(0.56)	0.000	0.061
Any infection	7925(22.16)	7310(18.19)	7817(17.62)	7547(15.52)	8289(14.95)	8295(14.66)	9153(15.93)	56336(16.65)	-0.010	0.020
>1 infection	466(1.30)	279(0.69)	144(0.32)	218(0.45)	236(0.43)	313(0.55)	343(0.60)	1999(0.59)	-0.010	0.204

Abbreviations: HIV, human immunodeficiency virus; STD, sexually transmitted disease; CT, *Chlamydia trachomatis*; NG, *Neisseria gonorrhoeae*; MG, *Mycoplasmagenitalium*; TP, syphilis; HBV, hepatitis B virus; HCV, hepatitis C virus; ND, no data.

^a^Other infections = CT/NG/MG/TP/HBV/HCV

^b^ Any infection = HIV/CT/NG/MG/TP/HBV/HCV

^c^ Tested from October2016 to December2018.

^d^ Tested by RPR and TPPA from 2017 to 2018.

### Trends in STDs

From 2012 to 2018, the prevalence of CT, TP and HBV declined from 3.55% to 1.73% (β = -0.004, *P*_TREND_ = 0.001), 2.94% to 2.15% (β = -0.003, *P*_TREND_ = 0.014), and 13.43% to 12.01% (β = -0.004, *P*_TREND_ = 0), respectively. From 2016 to 2018, the prevalence of MG positivity among the infertile population during prepregnancy testing increased from 2.55% to 2.90% (β = 0.006, *P*_TREND_ = 0.009)(Table 2). The prevalence of any infection among the infertile population decreased from 22.16% to 16.65% (β = -0.01, *P*_TREND_ = 0.02). However, the variation in HIV prevalence was not significant during 2012-2018(β = 0.000, *P*_TREND_ = 0.907). Subgroup analysis stratified by sex indicated that HIV prevalence in 2013 peaked at 0.10% in infertile men and at 0.03% in infertile women and then decreased and stabilized at 0.02–0.03% in the ensuing years (Table 2).

### HIV-infected infertile couples

From 2012 to 2018, the characteristics of the HIV-infected infertile population had not shifted dramatically: women accounted for 32.56% of HIV cases in China, and the incidence rate for men was 2 times that for women(Table 3). Concordant infections were found in 4.65% of HIV-infected infertile couples (6/129). The prevalence of other infections was as follows: CT in 14 cases (10.85%) and TP in 12 cases (9.30%). HIV can infect people of all ages. The largest group affected (54.26%,70/129) were persons of reproductive age (30–39 years), which was consistent with the age distribution of population in this study. Overall, 87.60% of the HIV-infected individuals had a relatively low education level (high school or below). Only 37.98%(49/129) of HIV-positive patients engaged in high-risk behaviors, such as injecting drugs or having sex with casual nonspousal partners. Heterosexual transmission had become the predominant source (31.78%) of HIV infection in our study. All HIV-positive women discontinued treatment. Of the HIV-positive men, 45.98%(40/87) continued their assisted reproductive therapy with donor semen, where as the others discontinued treatment.

**Table 3:** Sociodemographic, relationship, and sexual history characteristics of HIV-infected populations in infertile couples in China from 2012–2018.

Characteristic	n(%)							
	2012 (n = 11)	2013 (n = 23)	2014 (n = 13)	2015 (n = 19)	2016 (n = 18)	2017 (n = 13)	2018 (n = 32)	2012–2018 (n = 129)
Sex								
Male	8(72.73)	17(73.91)	6(46.15)	11(57.89)	13(72.22)	8(61.54)	24(75.00)	87(67.44)
Female	3(27.27)	6(26.09)	7(53.85)	8(42.11)	5(27.78)	5(38.46)	8(25.00)	42(32.56)
Male/Female	2.67:1	2.83:1	0.86:1	1.38:1	2.60:1	1.60:1	3.00:1	2.07:1
Age,y								
<20	0	0	0	0	0	0	0	0
20–29	5(45.45)	8(34.78)	4(30.77)	10(52.63)	4(22.22)	0	9(28.13)	40(31.01)
30–39	4(36.36)	11(47.83)	7(53.85)	7(36.84)	11(61.11)	11(84.62)	19(59.38)	70(54.26)
40–49	2(18.18)	4(17.39)	2(15.38)	2(10.53)	3(16.67)	2(15.38)	4(12.50)	19(14.73)
≥50	0	0	0	0	0	0	0	0
Education								
Never attended school	0	0	0	0	0	0	0	0
Primary school	1(9.09)	0	0	0	0	0	0	1(0.78)
Junior middle school	6(54.55)	11(47.83)	4(30.77)	8(42.11)	11(61.11)	3(23.08)	5(15.63)	48(37.21)
Senior school/technical secondary school	3(27.27)	9(39.13)	8(61.54)	9(47.37)	6(33.33)	5(38.46)	24(75.00)	64(49.61)
College or above	1(9.09)	3(13.04)	1(7.69)	2(10.53)	1(5.56)	5(38.46)	3(9.38)	16(12.40)
Income(RMB/month)								
<5000*	8(72.73)	14(60.87)	9(69.23)	13(68.42)	11(61.11)	8(61.54)	18(56.25)	81(62.79)
≥5000	3(27.27)	9(39.13)	4(30.77)	6(31.58)	7(38.89)	5(38.46)	14(43.75)	48(37.21)
High risk behaviors								
Injecting drugs/drug users	1(9.09)	0	1(7.69)	1(5.26)	0	1(7.69)	2(6.25)	6(4.65)
Having sex with nonspousalpartners	4(36.36)	6(26.09)	7(53.85)	5(26.32)	6(33.33)	4(30.77)	9(28.13)	41(31.78)
Having sex with men	0	0	1(7.69)	0	0	0	1(3.13)	2(1.55)
STD related symptoms								
Yes	2(18.18)	3(13.04)	1(7.69)	1(5.26)	2(11.11)	0	1(3.13)	10(7.75)
No	9(81.82)	20(86.96)	12(92.31)	18(94.74)	16(88.89)	13(100)	31(96.88)	119(92.25)
Coinfection with HIV								
CT	2(18.18)	1(4.35)	1(7.69)	4(21.05)	0	2(15.38)	4(12.50)	14(10.85)
NG	0	0	0	0	1(5.56)	0	1(3.13)	2(1.55)
MG	NA	NA	NA	NA	0	3(NA)	4(NA)	7(5.43)
TP	1(9.09)	4(17.39)	0	2(10.53)	2(11.11)	1(7.69)	2(6.25)	12(9.30)
HBV	9(81.82)	15(65.22)	2(15.38)	4(21.05)	3(16.67)	0	4(12.50)	37(28.68)
HCV	1(9.09)	1(4.35)	0	1(5.26)	1(5.56)	1(7.69)	2(6.25)	7(5.43)
HIV-infected spouse	0	3(13.04)	0	1(5.26)	0	0	2(6.25)	6(4.65)

*From October 2018, the starting point of personal income tax increased from 3500 RMB to 5000 RMB per month in China.

### Risk factors associated with HIV

Among the infertile couples surveyed, 67.44% of the HIV-infected and 44.80% of the HIV-uninfected group were male (odds ratio (OR) = 2.552, 95% CI: 1.697–3.839). Age stratification by 10-year age groups revealed a peak (70/129,54.26%; 265/500,53.00%) in HIV incidence trends for 30–39 years of age in HIV-infected and HIV-uninfected group, respectively. Age is not significantly associated with HIV infection (OR = 1.070, *p* = 0.761; OR = 1.054, *p* = 0.866). Participants who had an education level of junior middle school or below had a significantly higher risk of HIV infection, with an OR of 2.878 (95% CI:1.545–5.360, *p* = 0.001)(Table 4). Because the coinfection had smaller sample sizes, we were not able to assess risk factor. Compared to HIV-uninfected infertile patients, HIV-infected group have a higher rate of co-infection events, with other infected population being more susceptible to HIV infection.

**Table 4:** Risk factors in HIV-infected or HIV-uninfected among infertile couples in Hunan, China.

Factors	HIV-infected(n = 129)	HIV-uninfected(n = 500)	*p*-value	OR(95% CI)
Sex				
Male	87(67.44)	224(44.80)	<0.001	2.552(1.697,3.839)
Female	42(32.56)	276(55.20)		1.00
Age group				
<20	0(0)	10(0.02)	0.761^a^	1.070(1.697,3.839) ^a^
20–29	40(31.01)	152(30.40)		
30–39	70(54.26)	265(53.00)	0.866	1.054(0.572,1.944)
40–49	19(14.73)	70(14.00)		1.00 ^b^
≥50	0(0)	3(0.60)		
Education				
Never attended school	0(0)	0(0)	0.001 ^c^	2.878(1.545,5.360) ^c^
Primary school	1(0.78)	0(0)		
Junior middle school	48(37.21)	116(23.20)		
Senior school/technical secondary school	64(49.61)	275(55.00)	0.126	1.585(0.878,2.863)
College or above	16(12.40)	109(21.80)		1.00
Income(yuan/month)				
<5000*	81(62.79)	282(56.40)	0.196	0.767(0.515,1.142)
≥5000	48(37.21)	218(43.60)		1.00
Coinfection				
CT	14(10.85)	2(0.40)	<0.001^d^	NA
NG	2(1.55)	0(0)	0.042 ^d^	NA
MG	7(5.43)	0(0)	<0.001^d^	NA
TP	12(9.30)	1(0.20)	<0.001^d^	NA
HBV	37(28.68)	3(0.60)	<0.001^d^	NA
HCV	7(5.43)	0(0)	<0.001^d^	NA

Abbreviations: OR, odds ratio; CI, confidence interval; NA, not available.

^a, b, c^These two or three subgroups had smaller sample sizes, which were further merged into one group.

^d^Fisher's exact test.

## Discussion

These data constitute the first report of HIV prevalence among the infertile population in Hunan, China. The overall prevalence of HIV during 2012–2018 in China was 0.04% (129/338432), a relatively low epidemic level, which is different from the reported 0.0046%(2/43274) among infertile couples in Royan[29],1.7% (4/229)among recruited couples in Ghana[30], and 16.8% of primary infertile couples (women, 24/135; men, 19/121) and 35.5% of secondary infertile couples (women,74/177; men,36/133) in Rwanda[31]. We believed that the relatively high prevalences in these studies are related to their small sample sizes. The HIV prevalence of the infertile population is lower than that of the general population in China (0.09%) and other key control provinces (Yunnan, Guangxi, Sichuan, Henan, Guangdong and Xinjiang) in the southwest[7, 18]. Overall, the distribution of the epidemic is unbalanced. The HIV-positive population was more frequently diagnosed with coinfection than the HIV-negative population (61.24%,79/129 in Table 3 vs. 0.59%,1999/338432 in Table 2, p<0.001).

From 2011 to 2017, the incidence of AIDS in China increased by 36,744 cases, with a growth rate of 179.68%[17]. We found no significant increase (β_TREND_ = 0) in the prevalence of HIV among sexually active infertile individuals in China from 2012 to 2018 orbetweenthe sexes. The current data for HIV rates in China suggested that the HIV/AIDS epidemic remains low overall due to the effective implementation of evidence-based comprehensive strategies nationwide.

In addition, our study further explored risk factors for HIV infection. We found no significant differences in HIV-prevalence by age and income. HIV prevalence was significantly higher in men than in women, which also was observed with decreasing education level. Our results showed that 87.60% of the HIV-infected population had a low level of education. This may indicate that their perceptions and understanding of HIV risk are low. Those with better education are more knowledgeable about HIV and have more access to comprehensive sex education and treatment. Indeed, HIV/AIDS education programs commonly target adolescents and younger adults, and prevention programs should also be extended to middle-aged infertile adults. However, the self-perceived risk of HIV was low among all participants in this study. Lack of or low risk awareness of HIV may drive the low HIV testing rate. Only 4 participants admitted to having been tested for HIV in the last year. Thus, prevention strategies may need to be tailored to different age and risk groups, including teaching HIV symptoms and long-term risks, tests and treats.

Theoretically, due to physiological characteristics and social status and other factors, women are more likely to be infected with HIV. However, the ratio of men to women living with HIV in China was 9:1 in 1990–1995. By 2018, the characteristics of the HIV-infected population had shifted dramatically: the incidence rate for men was only 3 times that of women, as reported by National Health Care Commission. In our study, the proportion of men and women infected with HIV in 2012–2018 was 2.07:1, which was close to the data from 2018 mentioned in the literature. The following reasons may explain why the proportion of HIV prevalence between the sexes has changed so much: (1) the previously demonstrated increased vulnerability of women to HIV infection[32]. There is a broad consensus that male-to-female transmission is more efficient than female-to male transmission[33, 34]; (2) increased numbers of FSWs[13, 35]; (3) increased frequency of nonspousalsexual relationships[36]; (4) increased marriage of homosexualmales with women[13, 37]; and (5) increased high-risk sexual behavior among women[38].

Most STDs (98.87%) were asymptomatic in our study. Many people who experienced mild STD symptoms tended to buy antibiotics atpharmacies instead of admitting they had symptoms. To some extent, the approach used in this study may overestimate the asymptomatic rate of STD infections. Asymptomatic HIV was especially concerning. Without screening, a significant proportion of asymptomatic patients may not be aware of their HIV status. Because untreated infections persist, asymptomatic patients will not seek/delay seeking health services or adjust their risk practices in natural pregnancies, and the risk of contracting and then transmitting HIV to their spouse drastically increases. This increased coverage of IVF in China is indeed associated with a decreased risk of acquiring HIV. Thus, screening and treatment for HIV should be emphasized regardless of symptoms in the clinical setting. It is ethically appropriate to encourage HIV testing for all couples who want to have children not just for those who request infertility treatment[25]. In 2006, Centers for Disease Control and Prevention (CDC) released the Revised Recommendations for HIV Testing of Adults, Adolescents, and Pregnant Women in Health-Care Settings, which recommend screening for HIV (i.e., testing at least once, regardless of clinical signs or symptoms) for all persons aged 13–64 years[39]. This may help guide clinical practice and health policy to more effectively reduce the global burden of infertility.

There are a number of limitations to this study. First, the data were from our hospital. Although these data represent the infertile population in Hunan, China, the results may not be applicable to the wider infertile population in China. Second, the social stigma associated with HIV and the fact that disclosure of sensitive private self-reported information, such as sexual behavior, drug use, sex without a condom and number of sex partners, in face-to-face or telephone interviews are difficult for many patients, resulting in loss of data and information bias. Married people are less likely to report extramarital seethed, some HIV-infected patients are attracted to the famous sperm bank in our hospital for IVF with donor semen, possibly resulting in a high prevalence of HIV in the infertile population. However, given that HIV screening rates at non-STD clinics are much lower than those in our study, this factor unlikely had a large impact on our results. Nonetheless, such factors may lead to selection bias. Fourth, due to the cross-sectional nature of the study, no causality can be determined.

## Conclusions

Despite the aforementioned limitations, this study expands upon existing knowledge of HIV epidemics among the Chinese infertile population. This study demonstrates a need to improve public knowledge of HIV risk factors, though much work is needed for the popularization of prevention knowledge and change concepts. Therefore, targeted interventions should focus especially on populations with less education and high-risk behaviors. Moreover, routine HIV screening is urgently needed for all adults with high-risk behaviors, which may identify new HIV infections and increase opportunities for HIV care and prevention.

## Supporting information

S1 Checklist (DOCX)
