# Supplementary material for: HIV prevalence among 338,432 infertile individuals in Hunan, China, 2012-2018: A cross-sectional study
Source: PLoS One. 2020 Sep 10;15(9):e0238564. doi: 10.1371/journal.pone.0238564 (PMC7482923; doi:10.1371/journal.pone.0238564)
Supplement: S1 Checklist — (DOCX) [file pone.0238564.s001.docx]

STROBE Statement—checklist of items that should be included in reports of observational studies

|  | Item No. | Recommendation | Page  No. | Relevant text from manuscript |
| --- | --- | --- | --- | --- |
| **Title and abstract** | 1 | (*a*) Indicate the study’s design with a commonly used term in the title or the abstract | 1 | HIV prevalence among 338,432 infertile individuals in Hunan, China, 2012-2018: a cross-sectional study |
|  |  | (*b*) Provide in the abstract an informative and balanced summary of what was done and what was found | 2-3 | A cross-sectional hospital-based study was conducted to evaluate the prevalence of HIV/other infections. |
| Introduction | | | |  |
| Background/rationale | 2 | Explain the scientific background and rationale for the investigation being reported | 1 | HIV prevalence vary markedly among different risk groups in China, spreading from a high-risk population to the general population. Indeed, China is in a critical period of HIV/AIDS prevention and control, though data regarding HIV testing, infection and coinfection among infertile couples are lacking. |
| Objectives | 3 | State specific objectives, including any prespecified hypotheses | 1 | This study aimed to estimate HIV/AIDS prevalence to identify risk factors among infertile couples in Hunan, China. |
| Methods | | | |  |
| Study design | 4 | Present key elements of study design early in the paper | 2 | Cross-sectional hospital-based study.HIV/other STDs |
| Setting | 5 | Describe the setting, locations, and relevant dates, including periods of recruitment, exposure, follow-up, and data collection | 5 | TThis cross-sectional hospital-based study evaluated the prevalence of HIV/other STDs among 338,432 infertile couples who visited the outpatient department of the Reproductive and Genetic Hospital of CITIC Xiangya for prepregnancy examination between January 2012 and December 2018. We extracted relevant demographic and clinical information from all medical record data on January 2019.STD-related symptoms were measured by asking whether the participants experienced any of the listed symptoms in the last year.HIV-positive participants were administered a structured questionnaire in privates. |
| Participants | 6 | (*a*) *Cohort study*—Give the eligibility criteria, and the sources and methods of selection of participants. Describe methods of follow-up  *Case-control study*—Give the eligibility criteria, and the sources and methods of case ascertainment and control selection. Give the rationale for the choice of cases and controls  *Cross-sectional study*—Give the eligibility criteria, and the sources and methods of selection of participants | 6 | Infertile individuals who visited the outpatient department of Reproductive and Genetic Hospital of CITIC Xiangya between 2012 and 2018 were included. The following exclusion criteria were applied: duplicate records; incomplete information records; significant abnormality records; and refusal of counseling and testing for HIV/other infections, lack of completion of all study procedures. |
|  |  | (*b*) *Cohort study*—For matched studies, give matching criteria and number of exposed and unexposed  *Case-control study*—For matched studies, give matching criteria and the number of controls per case |  |  |
| Variables | 7 | Clearly define all outcomes, exposures, predictors, potential confounders, and effect modifiers. Give diagnostic criteria, if applicable | 7 | Those with confirmed positive tests were referred to Hunan Provincial Center for Disease Prevention and Control for further confirmation and reports.In this study, subjects with HbsAg-positive results were considered to have HBV infection.HCV antibody testing was performed with an ELISA diagnostic kit to detect antibodies against HCV. Subjects with positive results for both RPR and TPPA were considered to have a current TP infection. Male meatus urinarius secreta and female cervical secreta were collected for CT and NG testing by colloidal gold assays, culture and Gram staining with chocolate agar plate culture. Semen and vaginal swab specimens were tested via real-time probe simultaneous amplification and testing (SAT) for MG. The target gene 16S ribosomal RNA (rRNA) of MG was isolated and reverse transcribed to generate cDNA fragments. The results were interpreted as positive when the cycle threshold (Ct) was ≤35 and an absorption peak was observed in the melting curve. |
| Data sources/ measurement | 8* | For each variable of interest, give sources of data and details of methods of assessment (measurement). Describe comparability of assessment methods if there is more than one group | *8* | Analyses are descriptive, as performed using IBM SPSS Statistics. Baseline characteristics are presented as frequencies (%) for categorical data. The prevalence of HIV/STD infection with 95% confidence intervals (CI) was calculated. We calculated linear trends in prevalence for 2012-2018 using bivariate linear regression. Beta coefficients for year represent the average percentage point change (divided by 100) from one year to the next. Risk factors were assessed using chi square statistics for categorical variables. All of the tests were two-tailed, and p values <0.05 were interpreted as statistically significant. |
| Bias | 9 | Describe any efforts to address potential sources of bias | 5 | We analysis a large-scale samples. |
| Study size | 10 | Explain how the study size was arrived at | 5 | Total of 338,432 infertile couples |

Continued on next page

| Quantitative variables | 11 | Explain how quantitative variables were handled in the analyses. If applicable, describe which groupings were chosen and why | 5 | The width of the defined age groups was designed to be equal among the five age groups (<20, 20-29, 30-39, 40-49 and ≥50 years of age).Baseline characteristics are presented as frequencies (%) for categorical data. The prevalence of HIV/STD infection with 95% confidence intervals (CI) was calculated. We calculated linear trends in prevalence for 2012-2018 using bivariate linear regression. Beta coefficients for year represent the average percentage point change (divided by 100) from one year to the next. Risk factors were assessed using chi square statistics for categorical variables. |
| --- | --- | --- | --- | --- |
| Statistical methods | 12 | (*a*) Describe all statistical methods, including those used to control for confounding | 8 | Bivariate linear regression;risk factors were assessed using chi square statistics for categorical variables |
|  |  | (*b*) Describe any methods used to examine subgroups and interactions | 8 | chi square statistics |
|  |  | (*c*) Explain how missing data were addressed | 6 | exclude missing data |
|  |  | (*d*) *Cohort study*—If applicable, explain how loss to follow-up was addressed  *Case-control study*—If applicable, explain how matching of cases and controls was addressed  *Cross-sectional study*—If applicable, describe analytical methods taking account of sampling strategy |  | Not applicable. |
|  |  | (*e*) Describe any sensitivity analyses |  | Not applicable. |
| Results | | | | |
| Participants | 13* | (a) Report numbers of individuals at each stage of study—eg numbers potentially eligible, examined for eligibility, confirmed eligible, included in the study, completing follow-up, and analysed | 9 | Total of 338,432 infertile individuals were included.Only 1.13% of the participants (382/338432) reported STD signs and symptoms suggesting genital tract infection. |
|  |  | (b) Give reasons for non-participation at each stage | 6 | Duplicate records; incomplete information records; significant abnormality records; and refusal of counseling and testing for HIV/other infections, lack of completion of all study procedures. |
|  |  | (c) Consider use of a flow diagram |  | Not applicable. |
| Descriptive data | 14* | (a) Give characteristics of study participants (eg demographic, clinical, social) and information on exposures and potential confounders | 15 | These data represent the infertile population in Hunan, China, the results may not be applicable to the wider infertile population in China. The social stigma associated with HIV and the fact that disclosure of sensitive private self-reported information in face-to-face or telephone interviews are difficult for many patients, resulting in loss of data and information bias. Some HIV-infected patients are attracted to our hospital for IVF with donor semen, possibly resulting in a high prevalence of HIV in the infertile population. |
|  |  | (b) Indicate number of participants with missing data for each variable of interest | 9 | Only 1.13% of the participants (382/338432) reported STD signs and symptoms suggesting genital tract infection. |
|  |  | (c) *Cohort study*—Summarise follow-up time (eg, average and total amount) |  | Not applicable. |
| Outcome data | 15* | *Cohort study*—Report numbers of outcome events or summary measures over time |  | Not applicable. |
|  |  | *Case-control study—*Report numbers in each exposure category, or summary measures of exposure |  | Not applicable. |
|  |  | *Cross-sectional study—*Report numbers of outcome events or summary measures | *9* | Not applicable. |
| Main results | 16 | (*a*) Give unadjusted estimates and, if applicable, confounder-adjusted estimates and their precision (eg, 95% confidence interval). Make clear which confounders were adjusted for and why they were included | 9 | The overall prevalence rates of HIV, CT, NG, MG, TP, HBV and HCV antibody positivity in this study were 0.04%, 1.73%, 0.05%, 2.60%, 2.15%, 12.01% and 0.56%, respectively. Only 1.13% of the participants (3824/338432) reported STD signs and symptoms suggesting genital tract infection. Most infertile participants in this study did not perceive any personal risk for HIV (99.99%, 338,426/338,432). Only 4 participants admitted to having been tested for HIV in the past year, and 2 infertile men reported prior history of HIV, using in vitro fertilization with donor semen (AID) in our hospital. |
|  |  | (*b*) Report category boundaries when continuous variables were categorized | 5 | The width of the defined age groups was designed to be equal among the five age groups (<20, 20-29, 30-39, 40-49 and ≥50 years of age). |
|  |  | (*c*) If relevant, consider translating estimates of relative risk into absolute risk for a meaningful time period | 11 | Among the infertile couples surveyed, 67.44% of the HIV-infected and 44.80% of the HIV-uninfected group were male (OR= 0.392, 95%CI: 0.260-0.589). Participants who had an education level of senior school or below had a significantly higher risk of HIV infection, with an OR of 0.508 (95% CI: 0.289-0.894) |

Continued on next page

| Other analyses | 17 | Report other analyses done—eg analyses of subgroups and interactions, and sensitivity analyses | 10 | Subgroup analysis stratified by sex indicated that HIV prevalence in 2013 peaked at 0.10% in infertile men and at 0.03% in infertile women and then decreased and stabilized at 0.02-0.03% in the ensuing years.Age stratification by 10-year age groups revealed a peak (70/129,54.26%) in HIV incidence trends for 30–39 years of age, which was statistically significant compared with the other age groups (p<0.001). Overall, 87.60% of the HIV-infected individuals had a relatively low education level (high school or below). Only 37.98% (49/129) of HIV-positive patients engaged in high-risk behaviors, such as injecting drugs or having sex with casual nonspousal partners. |
| --- | --- | --- | --- | --- |
| Discussion | | | | |
| Key results | 18 | Summarise key results with reference to study objectives | 13 | We found no significant increase (βTREND=0) in the prevalence of HIV among sexually active infertile individuals in China from 2012 to 2018 or between the sexes. The proportion of men and women infected with HIV in 2012-2018 was 2.07:1.Among the infertile couples surveyed, 67.44% of the HIV-infected and 44.80% of the HIV-uninfected group were male . Participants who had an education level of senior school or below had a significantly higher risk of HIV infection, with an OR of 0.508. |
| Limitations | 19 | Discuss limitations of the study, taking into account sources of potential bias or imprecision. Discuss both direction and magnitude of any potential bias | 15 | First, the data were from our hospital. Although these data represent the infertile population in Hunan, China, the results may not be applicable to the wider infertile population in China. Second, the social stigma associated with HIV and the fact that disclosure of sensitive private self-reported information in face-to-face or telephone interviews are difficult for many patients, resulting in loss of data and information bias. Third, some HIV-infected patients are attracted to our hospital for IVF with donor semen, possibly resulting in a high prevalence of HIV in the infertile population. However, given that HIV screening rates at non-STD clinics are much lower than those in our study, this may be unlikely to have had a large impact on our results. Nonetheless, such factors may lead to selection bias. Fourth, due to the cross-sectional nature of the study, no causality can be determined. |
| Interpretation | 20 | Give a cautious overall interpretation of results considering objectives, limitations, multiplicity of analyses, results from similar studies, and other relevant evidence | 13 | We found no significant increase in the prevalence of HIV among sexually active infertile individuals in China from 2012 to 2018 or between the sexes.In our study, the proportion of men and women infected with HIV in 2012-2018 was 2.07:1, which was close to the data in 2018 mentioned in the literature. The following reasons may explain why the proportion of HIV prevalence between the sexes has changed so much. (1) The previously demonstrated increased vulnerability of women to HIV infection. (2) Increased numbers of female sex workers (FSW). (3) Increased frequency of nonspousal sexual relationships. (4) Increased marriage of homosexual males with women. (5) Increased high-risk sexual behavior among women.Our results showed that 87.60% of the HIV-infected population had a low level of education. This may indicate that their perceptions and understanding of HIV risk are low. |
| Generalisability | 21 | Discuss the generalisability (external validity) of the study results | 15 | This study expands upon existing knowledge of HIV epidemics among the Chinese infertile population. This study demonstrates a need to improve public knowledge of HIV risk factors, though much work is needed for the popularization of prevention knowledge and change concept. Therefore, targeted interventions should focus especially on populations with less education and high-risk behaviors. Moreover, routine HIV screening is urgently needed for all adults with high-risk behaviors. |
| Other information | |  | | |
| Funding | 22 | Give the source of funding and the role of the funders for the present study and, if applicable, for the original study on which the present article is based | 16 | This study was supported by the Clinical Scientific Research Special Grant from the Chinese Medical Association (grant number 17020410710,Wei-Na Li) and the Fundamental Research Funds for the Central Universities of Central South University(grant number 502211905, Rui Ding).Wei-Na Li conceived and designed the study. Wei-Na Li, Rui Ding collected the data and performed the analysis and drafted the paper. |

*Give information separately for cases and controls in case-control studies and, if applicable, for exposed and unexposed groups in cohort and cross-sectional studies.

**Note:** An Explanation and Elaboration article discusses each checklist item and gives methodological background and published examples of transparent reporting. The STROBE checklist is best used in conjunction with this article (freely available on the Web sites of PLoS Medicine at http://www.plosmedicine.org/, Annals of Internal Medicine at http://www.annals.org/, and Epidemiology at http://www.epidem.com/). Information on the STROBE Initiative is available at www.strobe-statement.org.
